# Adsorption of Phenol from Aqueous Solution Using *Lantana camara*, Forest Waste: Kinetics, Isotherm, and Thermodynamic Studies

**DOI:** 10.1155/2014/201626

**Published:** 2014-10-28

**Authors:** C. R. Girish, V. Ramachandra Murty

**Affiliations:** ^1^Department of Chemical Engineering, Manipal Institute of Technology, Manipal 576104, India; ^2^Department of Biotechnology, Manipal Institute of Technology, Manipal 576104, India

## Abstract

The present work investigates the potential of *Lantana camara*, a forest waste, as an adsorbent for the phenol reduction in wastewater. Batch studies were conducted with adsorbent treated with HCl and KOH to determine the influence of various experimental parameters such as pH, contact time, adsorbent dosage, and phenol concentration. The experimental conditions were optimized for the removal of phenol from wastewater. Equilibrium isotherms for the adsorption of phenol were analyzed by Freundlich, Langmuir, Temkin, and Dubinin-Radushkevich isotherm models. Thermodynamic parameters like the Gibbs free energy (Δ*G*°), enthalpy (Δ*H*°), and entropy (Δ*S*°) were also determined and they showed that the adsorption process was feasible, spontaneous, and exothermic in the temperature range of 298–328 K. The kinetic data were fitted with pseudo-second-order model. The equilibrium data that followed Langmuir model with the monolayer adsorption capacity was found to be 112.5 mg/g and 91.07 mg/g for adsorbent treated with HCl and KOH, respectively, for the concentration of phenol ranging from 25 to 250 mg/L. This indicates that the *Lantana camara* was a promising adsorbent for the removal of phenol from aqueous solutions.

## 1. Introduction

Phenol is one of the crucial pollutants released from the wastewater originating from the chemical industries like pulp and paper, gas and coke manufacturing, tanning, textile, plastics, rubber, pharmaceutical industries, ferrous industries and petroleum refinery and its substantial concentration in wastewater is listed in Table 1 [1, 2].

Phenol causes adverse effects on public health and environment. As per United States Environmental Protection Agency (USEPA) the allowable concentration of phenol in surface water should be less than 1.0 *μ*g/L [3]. Phenolic compounds are very harmful even at very low concentrations due to their toxic and carcinogenic properties. They cause damage to the eyes and the tissue under the skin, inhalation, or ingestion, can damage the respiratory and gastrointestinal tracts, and can lead to genetic damage [4]. Phenol is designated as the 11th of the 126 priority pollutants by the United States Environmental Protection Agency [5]. Therefore, it is an indispensable requirement to treat the phenol from industrial effluents before discharging into the water stream.

Various treatment methods such as biodegradation, biosorption, membrane separation, pervaporation, solvent extraction, distillation, and adsorption using activated carbon prepared from various precursors had been reviewed by Girish and Ramachandra Murty [6] to remove phenolic compounds from aqueous solution. Adsorption on activated carbon is the most widely used and the most effective adsorbent in treating phenolic wastewaters. It was elucidated that activated carbon can be formed from any carbonaceous solid precursor material. The desired physical and chemical properties that can be attained in the final activated carbon can be managed by the selection of the starting material. A wide range of diverse materials have been investigated as potential adsorbents for phenol removal in wastewater treatment. The prominent among them comprises silica gel [7, 8], activated alumina [9, 10], zeolites [4, 11], and red mud [12, 13].

But the drawback associated with the above materials is high cost and being nonrenewable in nature, which is a major economic consideration. This has excited a growing research interest in the production of activated carbon from locally available agricultural materials, especially for application concerning wastewater treatment [14]. Girish and Murty [15] reviewed the various agricultural by-products found to be suitable precursors for production of activated carbon. Mohd Din et al. [16] described that the biomass obtained from these materials is cheaper, renewable, and abundantly available. So an attempt has been made to use agricultural waste materials as an adsorbent for reducing the pollutant in wastewater.

A vast number of agricultural materials have been used as adsorbents for the removal of phenolic compounds from wastewater. These include date stone [17],* Tamarindus indica* [18], vegetal cord [19], banana peel [20], palm seed coat [21], oil palm empty fruit bunch [22], date pit [23], black stone cherries [24], vetiver roots [25], sugarcane bagasse [26], and* Luffa cylindrica* [27]. All these materials provide an alternative to conventional sources, which are prospective raw materials for activated carbon production. Also, using these agricultural materials for adsorbent preparation brings the solution to the problem of handling wastes [16].

In the process of quest for new agricultural wastes as precursor for adsorbent, attempts have been made to produce adsorbent from dry stem of lantana trees by the chemical treatment process.* Lantana camara* is a poisonous weed that has been expanded in many regions of the world and it poses major threats to ecosystem [28]. The lantana stem was collected from the tropical moist deciduous forests, that is, eastern side of Western Ghats, Coorg region, Karnataka, India. In this study, the potential of chemically treated carbon from lantana barks was studied for the removal of phenol from aqueous solution.

A systematic study of the adsorption of phenol on chemically treated lantana material was reported. It also addresses the batch experiments conducted to study the effect of process variables such as pH, adsorbent dosage, initial phenol concentrations, and temperature on adsorption. The optimum experimental conditions were determined and thermodynamic studies were carried out to determine the nature of the adsorption process. From the literature, it is understood that the adsorption of phenol can be by three possible mechanisms: the *π*-*π* dispersion interaction, the hydrogen bonding formation, and the electron donor-acceptor complex mechanism [29–31]. Therefore, in order to understand the above mechanisms, different adsorption isotherms (Langmuir, Freundlich, Temkin, and Dubinin-Radushkevich isotherms) and kinetic models (pseudo-first, pseudo-second-order kinetics and intraparticle diffusion) were investigated to find out the most suitable models describing the experimental findings and the adsorbate-adsorbent interactions.

## 2. Materials and Methods

### 2.1. Materials

Phenol has a chemical formula C_6_H_5_OH with a molecular weight of 94 g/mol. Phenol of analytical grade (Merck India Ltd.) was used for the preparation of stock solution of concentration 1000 mg/L. The experimental solutions of concentration varying from 25 to 250 mg/L were prepared by diluting the stock solution to accurate proportions.

The other chemicals potassium hydroxide (Merck India Ltd., AR grade), potassium nitrate (Merck India Ltd., AR grade), zinc chloride (Merck India Ltd., AR grade), hydrochloric acid (SD Fine Chemicals, India, AR grade), sulphuric acid (SD Fine Chemicals, India, AR grade), and orthophosphoric acid (SD Fine Chemicals, India, AR grade) were used for the chemical treatment of carbon.

### 2.2. Preparation of Chemically Treated Carbon

The material* Lantana camara* was washed with distilled water for several times to remove all the foreign matters. The materials were initially dried in sunlight for 48 h, made into pieces, grounded to powder, and sieved to several particle sizes less than 0.075 mm. The proximate analysis of the raw powder was conducted to determine the fixed carbon, volatile matter, moisture, and ash content and is shown in Table 2. In order to improve the surface properties of the raw powder, various chemicals such as 3 M H_3_PO_4_, 3 M H_2_SO_4_, 3 M HCl, 3 M ZnCl_2_, 3 M KNO_3_, and 3 M KOH in a 1 : 1 ratio were added. Initially the powder was thoroughly mixed with the chemical overnight and then the slurry formed was dried at 105°C for 6 h in an oven. Then sufficient water was added to the mixture to remove the excess chemicals [32]. The process of washing with water was repeated 3-4 times until pH comes to 7 [33]. Then the powder was dried and stored for further studies. Initially the average particle size, pore volume, specific surface area, and removal capacity of the various chemically treated carbons and untreated carbon were investigated. Based on the preliminary results, as shown in Table 2, only the best two adsorbents treated with HCl and KOH were used for further analysis.

### 2.3. Characterization of Activated Carbon

The various properties were determined by the standard procedures [34]. The moisture content of the raw powder was found by heating a known weight of the sample in an air oven maintained at 110°C for about 60 min. Then the residue was ignited in a muffle furnace at 750°C for about 8 h and at 900°C for about 10 min to determine ash content and volatile matter, respectively. The average particle size was determined by particle size analyser (CILAS 1064, France). The surface area and total pore volume measurement of carbon were carried out using BET apparatus (Smart Instruments, India). The surface functional groups of carbon were estimated by Fourier transform infrared (FTIR) spectroscopy instrument (Shimadzu 8400S, Japan).

### 2.4. Adsorption Experiments

The influence of various experimental parameters such as pH, adsorbent dosage, contact time, and temperature on the adsorption of phenol from aqueous solutions was optimised in a batch mode of studies. The pH of solution was maintained at 2.5 to 12 by adding 0.1 M HCl or 0.1 M NaOH; the adsorbent dosages of both HCl and KOH treated carbon were varied from 0.25 to 3 g and the temperature varied from 298 to 328 K. After optimising the experimental parameters, the equilibrium and kinetic and thermodynamic studies were conducted in 250 mL conical flasks containing 200 mL phenol solution of different initial concentrations of 25, 50, 100, 150, 200, and 250 mg/L under the optimum conditions. The flasks were agitated in a temperature controlled shaker at 140 rpm and 298 K for 7 h and 8 h, respectively, for adsorbent treated with HCl and KOH, respectively, until equilibrium was established. After reaching the equilibrium time, the samples were taken from the flasks and filtered and the residual phenol concentrations were analysed using double beam UV spectrophotometer (UV-1700, Shimadzu, Japan). The samples were analysed spectrophotometrically at a wavelength of 270 nm by the aid of technical calibration curve prepared prior to the analysis [16]. The thermodynamic study was carried out in 250 mL conical flasks containing 200 mL phenol solution of different initial concentrations of 25, 50, 100, 150, 200, and 250 mg/L under the optimum conditions by varying temperature from 298 to 328 K. All sets of experiments were performed in duplicate under the optimum conditions and the mean values are presented. The error obtained was between 2.0 and 4.5%.

The amount of phenol adsorbed per gram of carbon (*q*_*e*_) was obtained using the following expression:

(1) $$\begin{array}{c} q_{e}=\frac{\;V*\left(C_{0}-C_{e}\right)}{1000M}, \end{array}$$

where *q*_*e*_ is the equilibrium adsorption capacity (mg/g), *V* is the solution volume (*L*), *C*_0_ (mg/L) is the initial phenol concentration, *C*_*e*_ (mg/L) is the equilibrium phenol concentration, and *M* is the weight of the carbon powder (g).

The percentage removal of the phenol is given by

(2) $$\begin{array}{c} \%\text{Removal}=\frac{C_{0}-C_{e}}{C_{0}}*100. \end{array}$$

### 2.5. Batch Kinetic Studies

The kinetic studies were carried out similar to those of equilibrium studies. The aqueous samples were collected at regular intervals and the concentrations of phenol solutions were similarly measured.

## 3. Results and Discussions

### 3.1. Characterisation of the Adsorbent

The proximate analysis of the raw powder which was carried out is shown in Table 2. The average particle size, pore volume, specific surface area, and removal capacity of the various carbons are shown in Table 3. Of the six adsorbent options, adsorbents treated with HCl and KOH were found to exhibit better results and were studied further. It was also inferred that the adsorption capacity of carbon is dependent on the porosity, specific surface area, and chemical composition.

**Figure 1:**
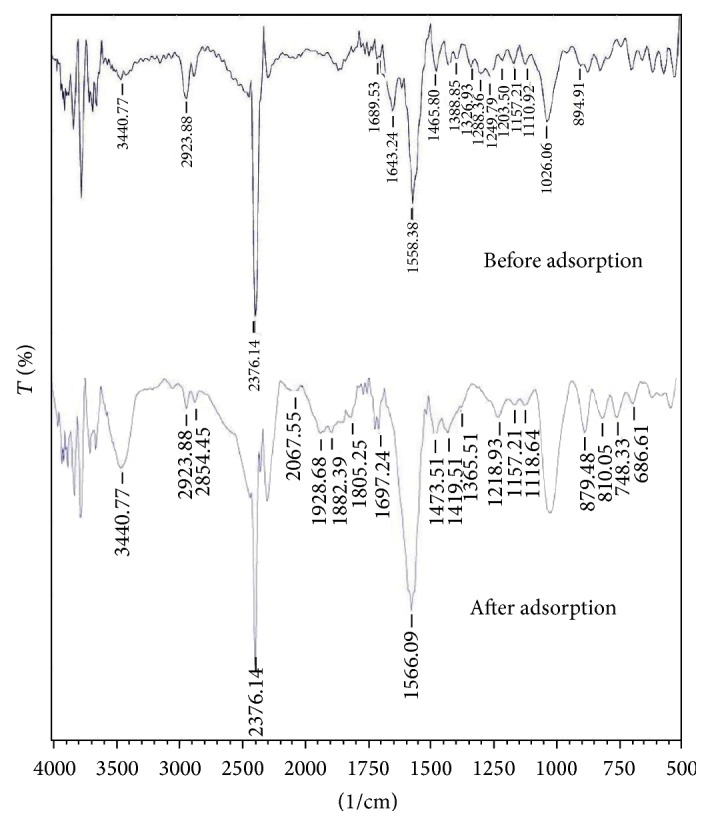
The FTIR spectra of adsorbent treated with HCl before and after phenol adsorption.

The FTIR spectra of adsorbent treated with HCl before and after phenol adsorption are shown in Figure 1. FTIR spectrum of carbon before phenol adsorption shows peaks at 2923 cm^−1^ due to O–H stretching in carboxylic group, the peak at 3620 cm^−1^ shows OH stretching of phenol group [35], the peak observed at 1203 cm^−1^ is C–O group attributed to alcohol, the band at 2376 cm^−1^ indicates the presence of C*≡*C of alkynes [36], and at 1558 cm^−1^ is ascribed to C=C aromatic ring stretching vibration [16]. The peak at 879 and 810 cm^−1^ was ascribed to C–H group of alkenes and at 1689 cm^−1^ due to C=O stretch of carboxylic acid [37]. The changes in peak of the spectral analysis for the various functional groups which could be the possible sites for phenol adsorption are shown in Table 4.

**Figure 2:**
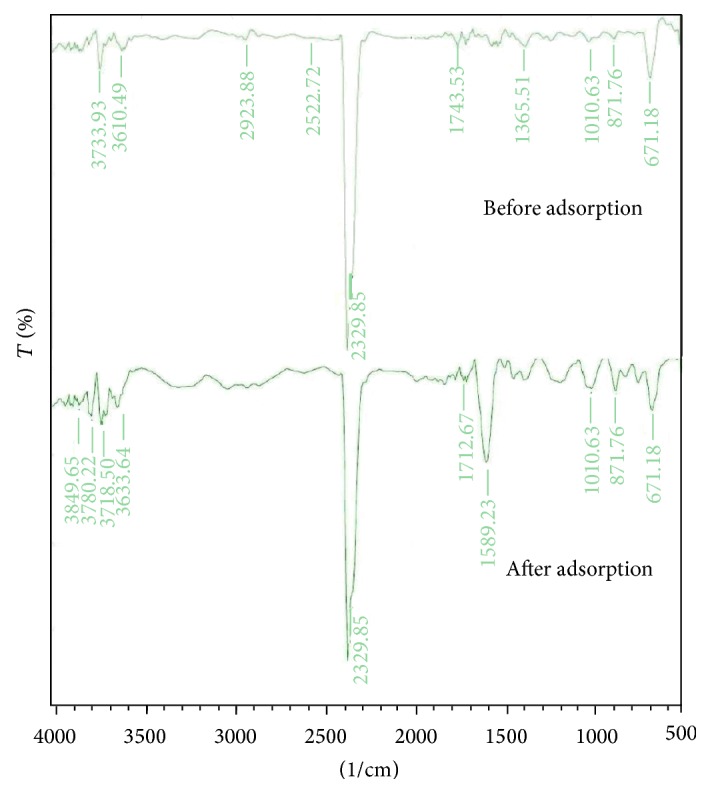
The FTIR spectra of adsorbent treated with KOH before and after phenol adsorption.

The adsorbent treated with KOH showed the FTIR spectrum as given in Figure 2. The peak at 3610 cm^−1^ is attributed to O–H stretching in phenol, the peak at 871 cm^−1^ is assigned to C–H of aromatic ring, the peak at 1365 cm^−1^ indicates the C–O bond of alcohol, and the band at 671 and 1010 cm^−1^ [38] is because of O–H stretching and C–O–C stretching of benzene derivative, respectively. The band at 2923 cm^−1^ shows C–H stretching of aliphatic group [39] and 2329 cm^−1^ is because of C*≡*C of alkynes, respectively [16]. The band obtained at 1743 cm^−1^ is because of stretching vibration of C=O in carboxyl group [40]. Similarly the changes in peak of the spectral analysis for the various functional groups are indicated in Table 5, which could be the possible sites for phenol adsorption.

### 3.2. Effect of pH

**Figure 3:**
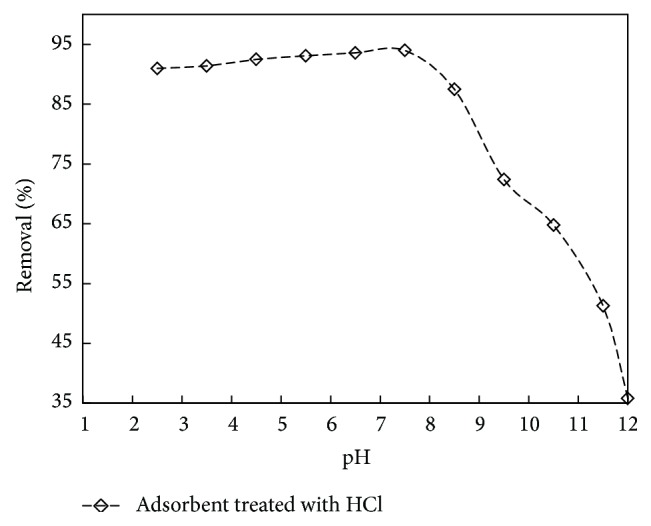
The effect of pH on % removal for adsorbent treated with HCl (initial concentration: 150 mg/L; volume: 200 mL; dosage: 0.75 g/L).

Because of the amphoteric nature of a carbon surface, the adsorption properties are influenced by the pH value of the solution. Phenol is a weak acid with acid dissociation value (pKa) of 9.8 and it dissociates into phenoxide ion when pH > pKa. At higher pH values the concentration of the negatively charged phenoxide ion increases and the electrostatic repulsions occur between the negative surface charge of the carbon and the phenoxide anions in solution. At lower pH values, phenolic compounds are present as the unionized acidic compounds [38, 41] and thereby increased the electrostatic attractions between the phenol and the adsorption sites. It can be observed from Figure 3 that, up to pH 7, the decrease in adsorption is gradual, which, however, drops drastically after pH 7 for adsorbent treated with HCl because of repulsion between negatively charged carbon surface and phenoxide ions.

**Figure 4:**
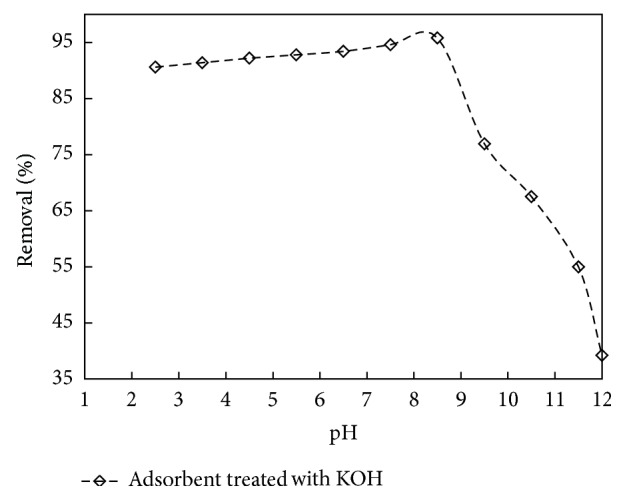
The effect of pH on % removal for adsorbent treated with KOH (initial concentration: 150 mg/L; volume: 200 mL; dosage: 1 g/L).

Similarly, from Figure 4, for adsorbent treated with KOH up to pH 8, there was gradual decrease in adsorption and thereafter it decreased drastically. The optimum pH value was found to be 7.5 and 8.5 for adsorbent treated with HCl and KOH, respectively. Similar results were reported in the literature [35, 42, 43].

### 3.3. Effect of Adsorbent Dosage

**Figure 5:**
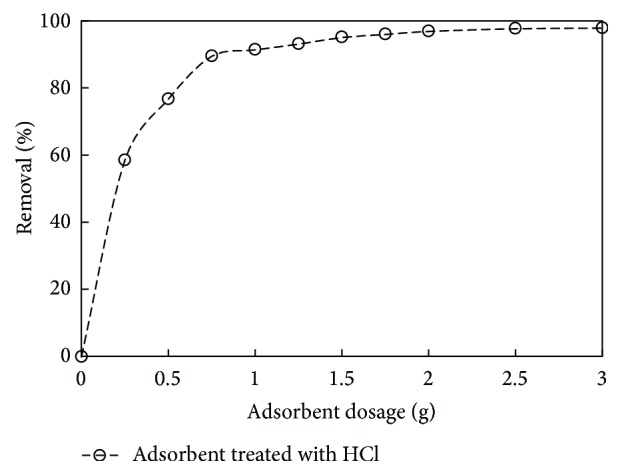
The effect of adsorbent dosage on % removal for adsorbent treated with HCl (initial concentration: 150 mg/L; volume: 200 mL; pH: 7.5).

**Figure 6:**
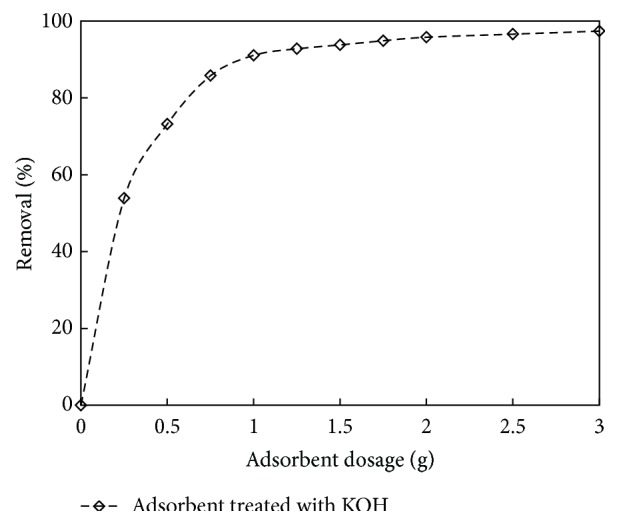
The effect of adsorbent dosage on % removal for adsorbent treated with KOH (initial concentration: 150 mg/L; volume: 200 mL; pH: 8.5).

To study the effect of adsorbent dose on phenol adsorption, the experiments were conducted at initial phenol concentration of 200 mg/L. Figures 5 and 6 show the effect of carbon dose on the removal of phenol. It was observed that the % removal increased with increase in adsorbent dose. After the equilibrium time, the removal was 58.6 to 89.6% for carbon dosage of 0.25 to 0.75 g/L for adsorbent treated with HCl and there was 53.9 to 91.1% removal for adsorbent dosage of 0.25 to 1 g/L for adsorbent treated with KOH, respectively. The increase in phenol removal is due to the increase of the available sorption surface and availability of more adsorption sites. It was also understood that, at higher carbon to solute concentration ratios, there is a higher sorption onto the adsorbent surface; thus it produces a lower solute concentration in the solution [44, 45]. It was found that the optimum carbon dosage was 0.75 g/L and 1 g/L for adsorbent treated with HCl and KOH, respectively. A similar observation was reported for removal of phenol from aqueous solution [21, 46].

### 3.4. Effect of Contact Time and Initial Concentration

**Figure 7:**
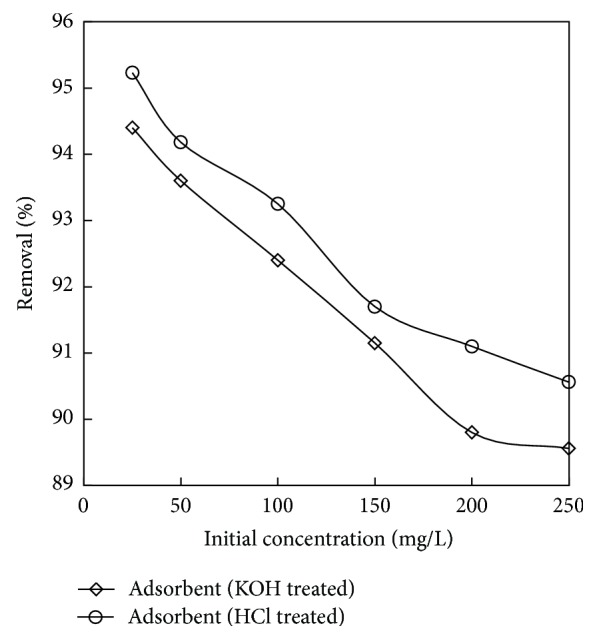
The plot showing the effect of initial concentration on % removal for the adsorbents (the initial concentration: 25 to 250 mg/L; dosage: 0.75 g/L for adsorbent (HCl) and 1 g/L for adsorbent (KOH); volume: 200 mL).

**Figure 8:**
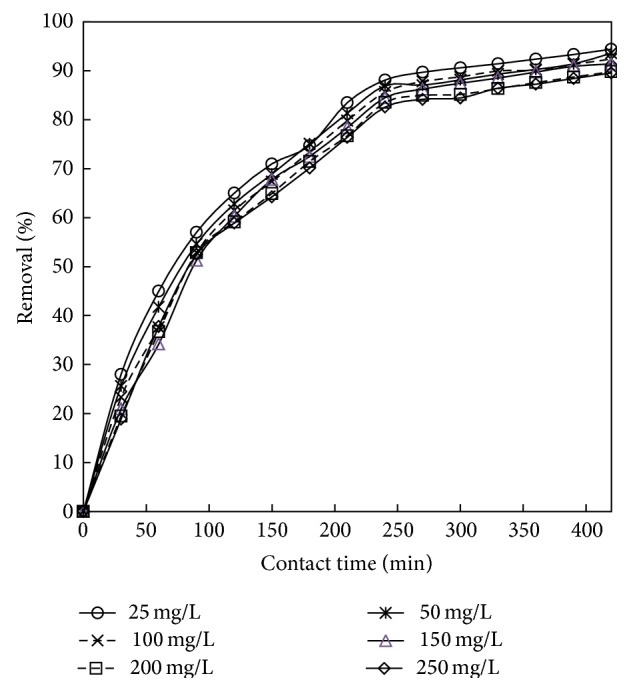
The plot showing the time v/s % removal for adsorbent treated with HCl (the initial concentration: 25 to 250 mg/L; dosage: 0.75 g/L; volume: 200 mL).

**Figure 9:**
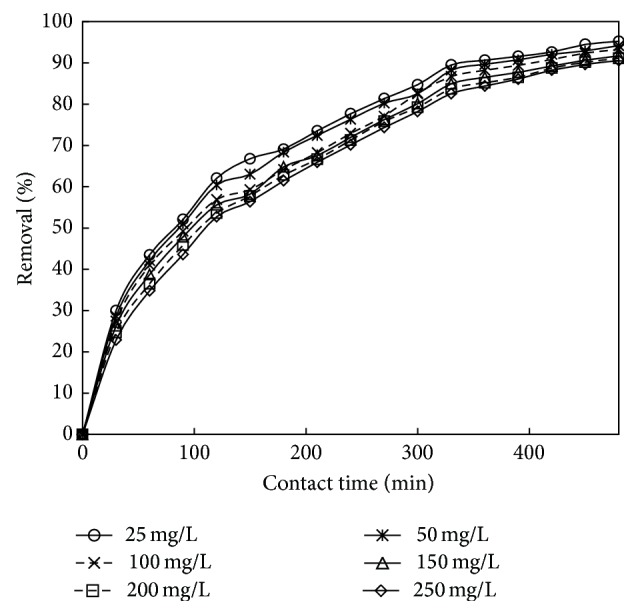
The plot showing the time v/s % removal for adsorbent treated with KOH (the initial concentration: 25 to 250 mg/L; dosage: 1 g/L; volume: 200 mL).

The initial concentration gives an important driving force required to overcome all mass transfer resistances of all molecules between the aqueous and solid phases [47]. The effect of initial phenol concentration on adsorption as shown in Figure 7 was studied in the range of 25–250 mg/L of the initial phenol concentrations under the optimized conditions. Figures 8 and 9 showed rapid adsorption of phenol in the period and thereafter the adsorption rate declined gradually and reached the equilibrium at about 6 h and 7 h for adsorbent treated with HCl and KOH. It was observed that, at the initial stage, adsorption rate is more, because of availability of more numbers of vacant sites. After a certain period of time, the rate of adsorption decreases due to accumulation of adsorbate in the vacant sites. It was also found from the figure that the increase in initial phenol concentration enhances the interaction between phenol and active sites in carbon surface, thus decreasing the % removal of phenol with increase in concentration. Therefore, an increase in initial concentration of phenol decreased the adsorption uptake of phenol. Similar type of results was reported in [48, 49].

### 3.5. Effect of Temperature

**Figure 10:**
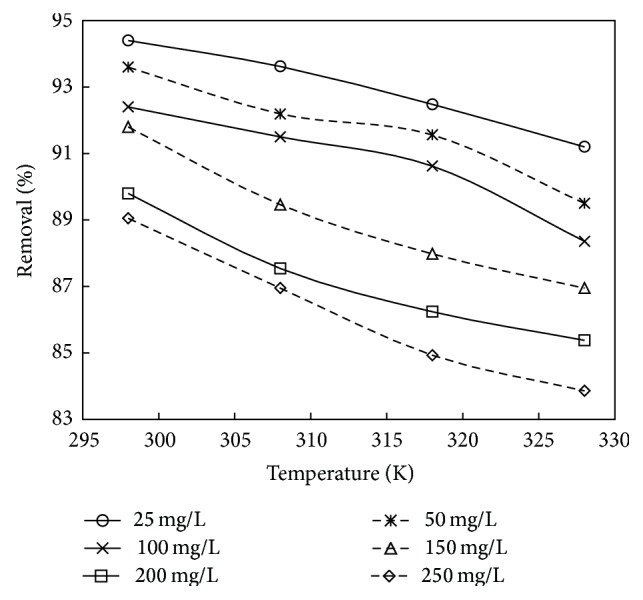
The plot showing the effect of temperature on % removal for adsorbent treated with HCl (the initial concentration: 25 to 250 mg/L; dosage: 0.75 g/L; volume: 200 mL).

**Figure 11:**
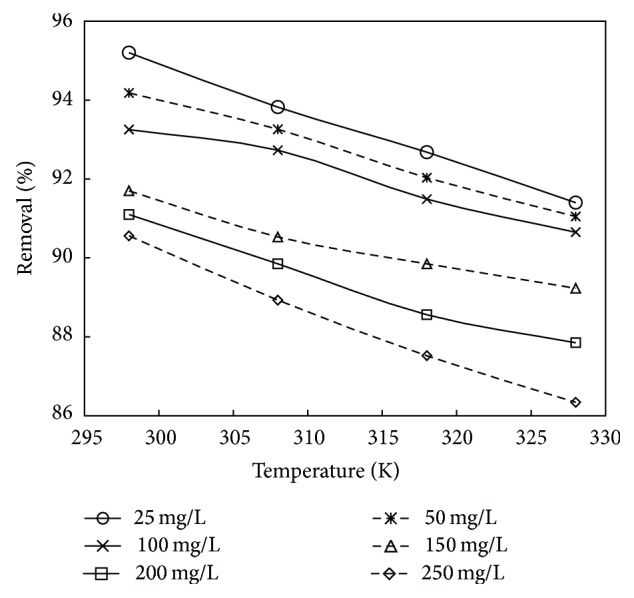
The plot showing the effect of temperature on % removal for adsorbent treated with KOH (the initial concentration: 25 to 250 mg/L; dosage: 1 g/L; volume: 200 mL).

The effect of temperature on the adsorption of phenol at various concentrations onto adsorbent treated with HCl and KOH is shown in Figures 10 and 11. Experiments were performed at different temperatures of 298, 308, 318, and 328 K. It can be observed from the figure that the % removal of phenol decreased with the increase in temperature from 298 to 328 K. This is probably due to the decreased chemical interaction between adsorbates and adsorbent indicating the exothermic nature of the adsorption process. Therefore, further adsorption experiments were performed at 298 K. Similar trend was obtained in works reported by [23, 35].

### 3.6. Isotherm Studies

Adsorption isotherm describes the relationship between the amount of a solute adsorbed and its concentration in the equilibrium solution at a constant temperature. Adsorption isotherm is important to understand the solute-adsorbent interactions and optimization of the use of adsorbents. Several models have been investigated in the literature to describe experimental data of adsorption isotherm. The equilibrium isotherms like Langmuir, Freundlich, Temkin, and Dubinin-Radushkevich isotherms were analysed in this study. A trial and error procedure was employed to estimate the above isotherms parameters by minimizing the error distribution between experimental data and predicted data using the solver add-in with Microsoft's Excel [49].

The Langmuir isotherm is based on the assumption that the adsorption process will take place uniformly within the adsorbent surface and with uniform distribution of energy level [50]. Once the adsorbate is attached on the site, no more adsorption takes place, showing that it is monolayer type of adsorption.

The Langmuir isotherm is

(3) $$\begin{array}{c} q_{e}=\frac{q_{m}K_{a}C_{e}}{1+K_{a}C_{e}}, \end{array}$$

where *q*_*m*_ (mg/g) and *K*_*a*_ (L/mg) are the Langmuir isotherm constants.

The Langmuir isotherm can also be expressed by a separation factor (*R*_*L*_), which is given by the equation

(4) $$\begin{array}{c} R_{L}=\frac{1}{\left(1+bC_{0}\right)}, \end{array}$$

where “*C*_0_” is the initial concentration of phenol in mg/L and “*b*” is the Langmuir constant in L/mg. The separation factor “*R*_*L*_” indicates the nature of the adsorption process [46] as given in Table 6.

The *R*_*L*_ values were found to be varying from 0.091933 to 0.503081 and 0.093897 to 0.508906 for adsorbent treated with HCl and KOH, respectively, showing that the adsorption process is favourable.

Freundlich isotherm [51] explains that the adsorption occurs on heterogeneous sites with nonuniform distribution of energy level and it also proposes reversible adsorption and possibility of adsorption on multilayers:

(5) $$\begin{array}{c} q_{e}=k_{f}{C_{e}}^{1/n}, \end{array}$$

where *q*_*e*_ is the amount of adsorbate adsorbed at equilibrium (mg/g), *C*_*e*_ is equilibrium concentration of the adsorbate (mg/L), *K*_*F*_ is Freundlich constant (mg/g) (L/mg)^1/*n*^  , and 1/*n* is adsorption intensity. The value of adsorption intensity shows the favourability of adsorption [52]. The value of *n* > 1 expresses favourable adsorption condition.

Temkin isotherm [53, 54] includes the influences of indirect adsorbate/adsorbate interactions on adsorption isotherms and explains that because of these interactions the heat of adsorption of all the molecules in the layer would decrease linearly with coverage.

The Temkin isotherm has been used in the following form:

(6) $$\begin{array}{c} q_{e}=B\ln AC_{e}, \end{array}$$

where *A* and *B* are Temkin isotherm constants.

The Dubinin-Radushkevich model [55] which is used to estimate the apparent free energy of adsorption has the following form:

(7) $$\begin{array}{c} q_{e}=q_{m}e^{{\beta \varepsilon }^{2}}, \end{array}$$

where *q*_*m*_ is the Dubinin-Radushkevich monolayer capacity (mg/g), *β* is a constant related to sorption energy, and *ε* is the Polanyi potential which is related to the equilibrium concentration in the following form:

(8) $$\begin{array}{c} \varepsilon =RT\ln\left(1+\frac{1}{C_{e}}\right), \end{array}$$

where *R* is the gas constant (8.314 J/mol K) and *T* is the absolute temperature. The constant *β* gives the mean free energy, *E*, of sorption per molecule of the sorbate and is given by the relation

(9) $$\begin{array}{c} E=\frac{1}{\sqrt{2\beta }}. \end{array}$$

**Figure 12:**
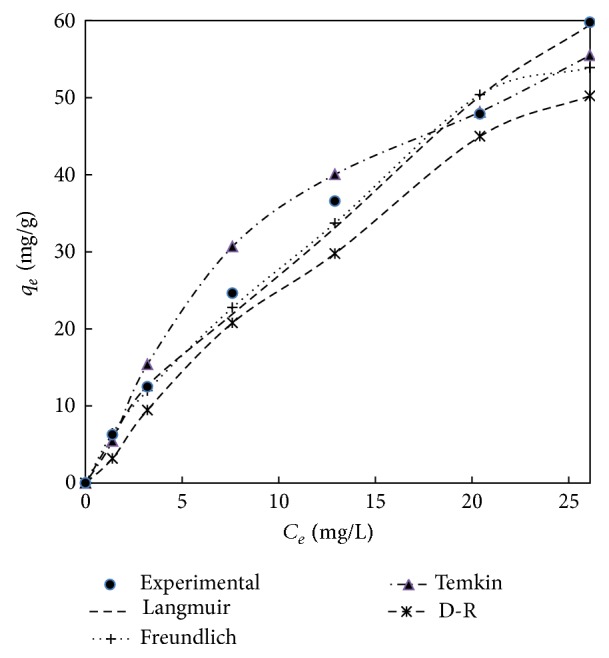
The comparison of various isotherm models for adsorbent treated with HCl.

**Figure 13:**
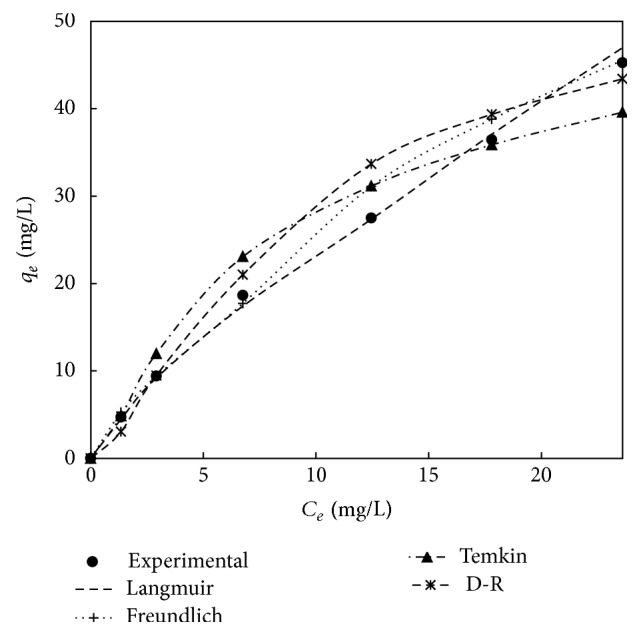
The comparison of various isotherm models for adsorbent treated with KOH.

The calculated isotherm constants by nonlinear method are represented in Tables 7 and 8 and the experimental equilibrium data and the predicted theoretical isotherms for the adsorption of adsorbent treated with HCl and KOH are shown in Figures 12 and 13. It can be observed from Figures 12 and 13 that Langmuir isotherm model is the best fit model when compared to Freundlich, Temkin, and Dubinin-Radushkevich isotherm model for adsorbent treated with both HCl and KOH. This is proved by the high value of correlation coefficient in case of Langmuir models compared to the other isotherm models. This concludes the fact that the adsorbent treated with both HCl and KOH follows monolayer adsorption on a surface that is homogenous. From the Tables 7 and 8, we can also get the maximum monolayer adsorption capacity (*q*_*m*_) of 112.5 mg/g and 91.07 mg/g for adsorbent treated with HCl and KOH, respectively.

The comparison of maximum monolayer adsorption capacity of phenol onto various agricultural adsorbents from the literature is presented in Table 9.

### 3.7. Thermodynamic Study

**Figure 14:**
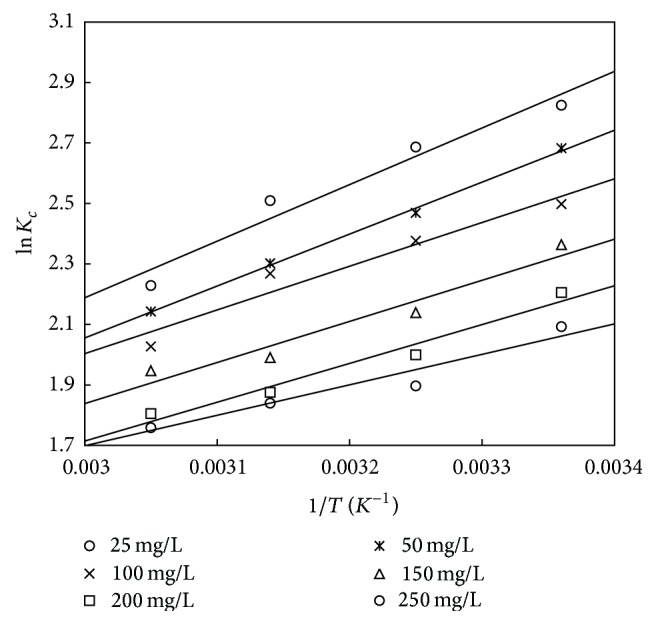
The van't Hoff plot for adsorbent treated with HCl.

**Figure 15:**
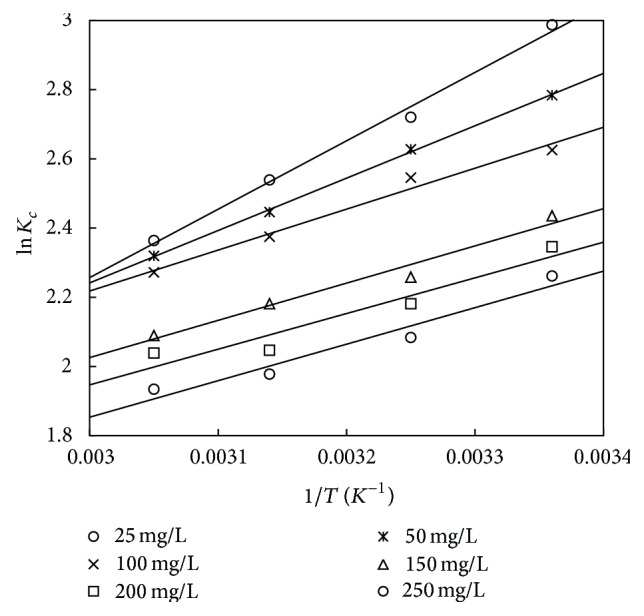
The van't Hoff plot for the adsorbent treated with KOH.

The feasibility of the adsorption process was estimated by the determination of thermodynamic parameters like free energy change (Δ*G*°), enthalpy (Δ*H*°), and entropy (Δ*S*°) which are calculated from the following equation:

(10) $$\begin{array}{c} K_{c}=\frac{C_{Ae}}{C_{e}},\\ \Delta G^{{}^{\circ}}=-RT\ln K_{c},\\ \Delta G^{{}^{\circ}}=\Delta H^{{}^{\circ}}-T\Delta S^{{}^{\circ}},\\ \ln K_{c}=\frac{{\Delta S}^{{}^{\circ}}}{R}-\frac{{\Delta S}^{{}^{\circ}}}{RT}, \end{array}$$

where *K*_*c*_ is the equilibrium constant, *C*_*e*_ is the equilibrium concentration in solution (mg/L), and *C*_*Ae*_ is the amount of phenol adsorbed on the adsorbent per liter of solution at equilibrium (mg/L). Δ*G*°, Δ*H*°, and Δ*S*° are changes in Gibbs free energy (kJ/mol), enthalpy (kJ/mol), and entropy (J/mol *K*), respectively, *R* is the gas constant (8.314 J/mol *K*), and *T* is the temperature (*K*). The values of Δ*H*° and Δ*S*° are determined from the slope and the interception of the plots of ln⁡*K*_*c*_ versus 1/*T* (Figures 14 and 15) and are shown in Tables 10 and 11. The negative values of Δ*G*° in the temperature range of 298 to 328 K show that the adsorption process is feasible and spontaneous. The negative value of Δ*H*° confirmed that the adsorption process is exothermic in nature. The negative value of Δ*S*° indicates the reduced randomness at the adsorbent/solution interface during the process of adsorption. It can also be observed that, at lower initial concentration, the values of Δ*G*° and Δ*H*° are more negative showing that the process is feasible and exothermic in nature. Similar nature of results was obtained in works of [35, 45, 55, 56].

### 3.8. Kinetics of the Adsorption

Adsorption kinetics has been examined to determine the adsorption mechanism. The various kinetic models reported that adsorption depends on the chemical nature of adsorbent, experimental conditions, and the mass transfer process. Therefore, in order to investigate the mechanism of present adsorption process and the rate-determining step, the different kinetic models like pseudo-first-order, pseudo-second-order, and intraparticle diffusion model were verified and the adsorption capacities were found.

**Figure 16:**
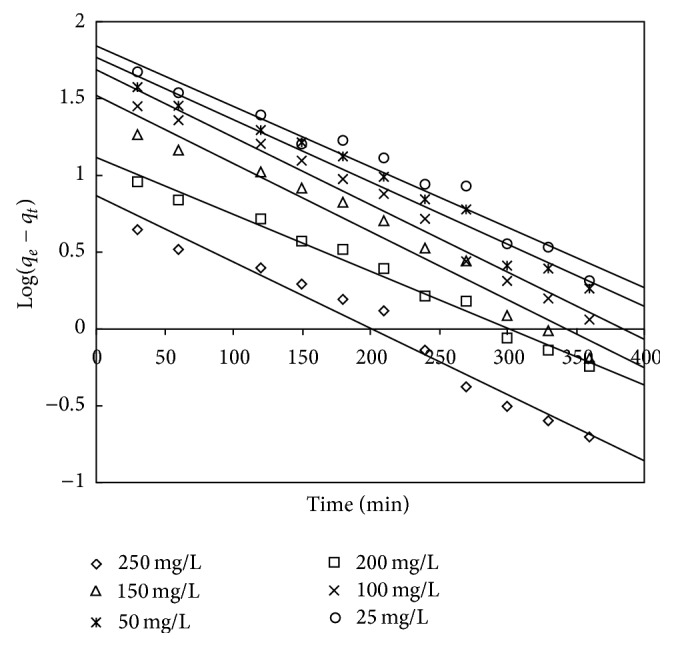
First-order kinetic plot for adsorbent treated with HCl.

**Figure 17:**
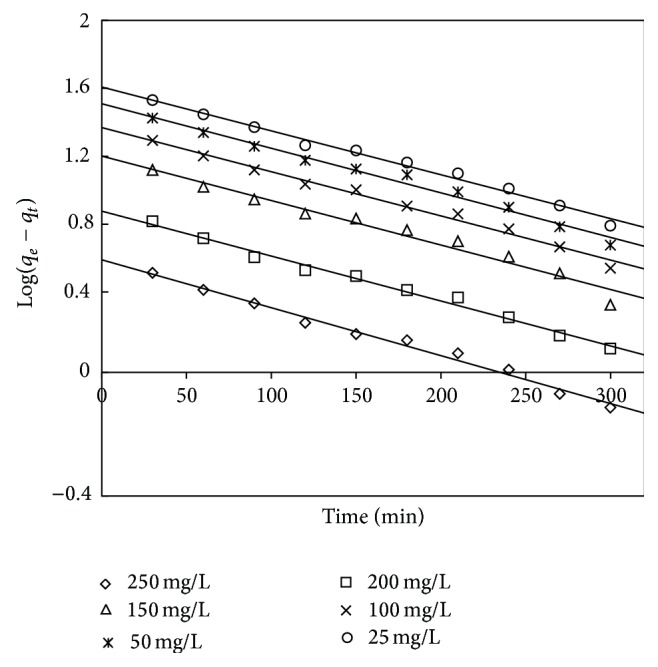
First-order kinetic plot for adsorbent treated with KOH.

The pseudo-first-order kinetic model in linear form is given by Lagergren [57]

(11) $$\begin{array}{c} \log\left(q_{e}-q_{t}\right)=\log q_{e}-\frac{k_{\text{ad}}}{2.303}t, \end{array}$$

where *q*_*t*_ is the adsorption capacity at time *t* (mg/g) and kad (min^−1^) is the rate constant of the pseudo-first-order adsorption. The rate constant *k*_ad_, adsorption capacity *q*_*e*_, and the correlation coefficients were obtained from the linear plots of log⁡(*q*_*e*_ − *q*_*t*_) versus *t* (as shown in Figures 16 and 17). The obtained values of *q*_*e*_ and *K*_ad_ and the corresponding linear regression correlation coefficient are shown in Tables 12 and 13. It was investigated that the correlation coefficients for the pseudo-first-order kinetic model for the adsorbent treated with both HCl and KOH are low. It was also observed that the values of calculated adsorption capacity and the experimental values deviated to a large extent, showing a poor fitting of experimental data to pseudo-first-order kinetic model.

**Figure 18:**
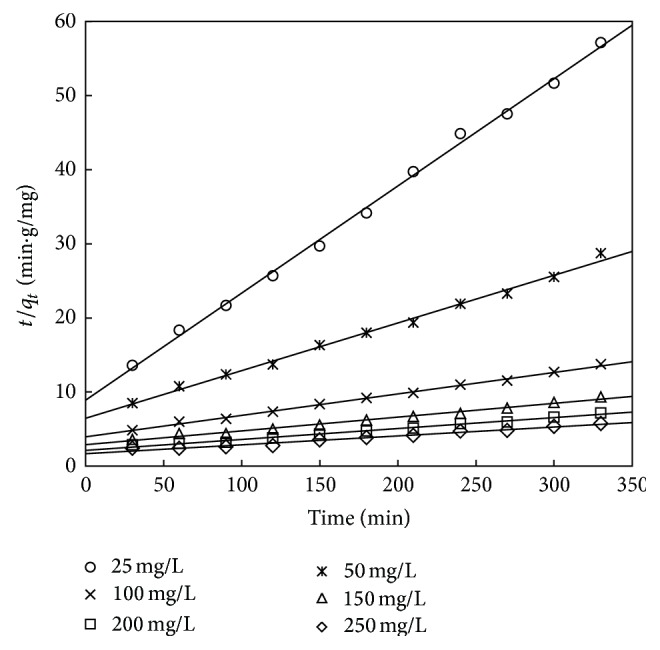
Second-order kinetic plot for adsorbent treated with HCl.

**Figure 19:**
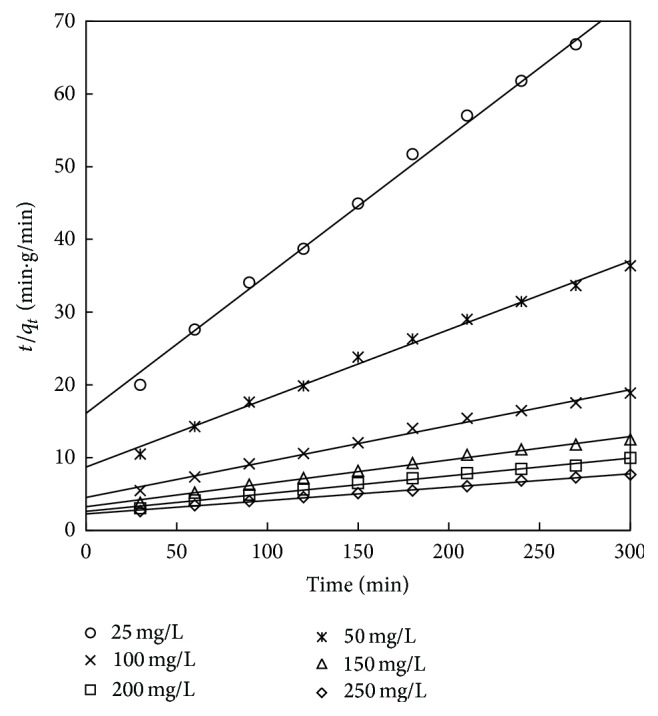
Second-order kinetic model for adsorbent treated with KOH.

The pseudo-second-order kinetic model is given by Ho [58]

(12) $$\begin{array}{c} \frac{t}{q_{t}}=\frac{1}{h}+\frac{1}{q_{e}}t, \end{array}$$

where *h* = *kq*_*e*_^2^ (mg g^−1 ^min^−1^) is the initial adsorption rate and *k* is the rate constant of pseudo-second-order model (g mg^−1 ^min^−1^). The values of *q*_*e*_, *k*, and *h* are obtained from the linear plot of *t*/*q*_*t*_ versus *t* shown in Figures 18 and 19. The values of experimental and calculated *q*_*e*_ along with correlation coefficient are presented in Tables 12 and 13. It can be observed from Tables 12 and 13 that, for the adsorbent treated with both HCl and KOH, the adsorption kinetics is better represented by pseudo-second-order kinetic model. This suggests that the rate controlling step of phenol onto adsorbent may be by chemisorption. From Tables 12 and 13, it was also found that the values of the rate constant *k* decreased with increasing initial phenol concentration for the pseudo-second-order model. This may be because of the reason that there is less competition for the active sites at lower concentration and high competition exists at the surface sites at higher concentrations. A similar result was reported for the adsorption of phenol from aqueous solution in banana peel [20], date pit carbon [23], coconut shell carbon, [16] and biomass material [41].

**Figure 20:**
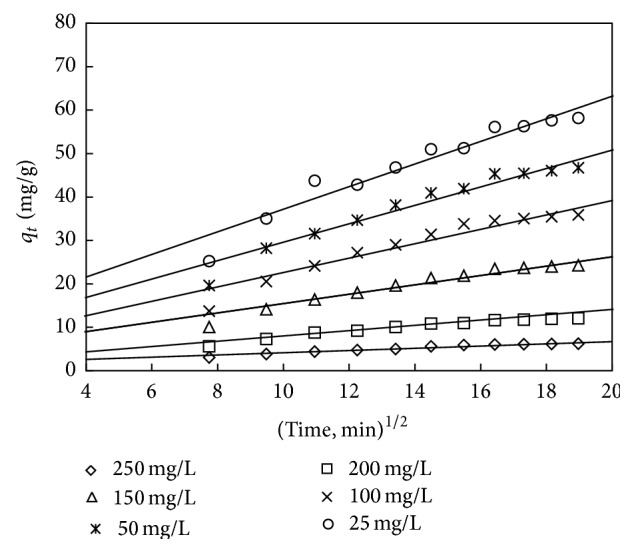
Intraparticle diffusion plot for adsorbent treated with HCl.

**Figure 21:**
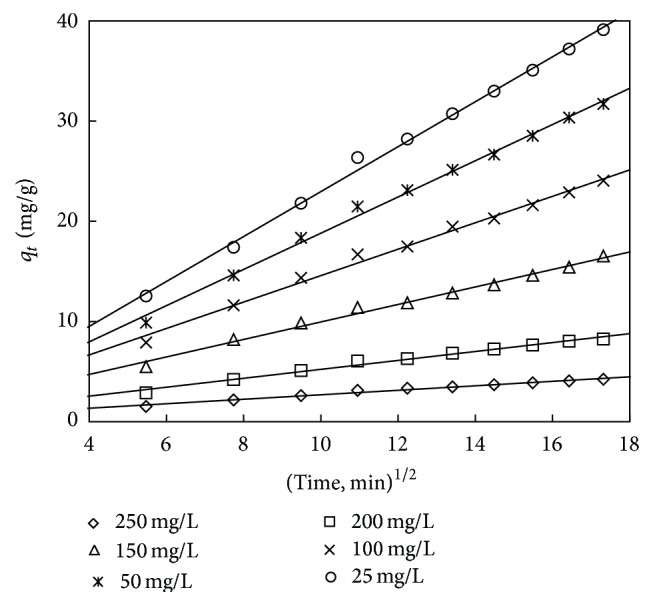
Intraparticle diffusion plot for adsorbent treated with KOH.

The kinetic data can be analysed using the Weber and Morris model [59] to understand the diffusion mechanism:

(13) $$\begin{array}{c} q_{t}=k_{p}t^{1/2}+c, \end{array}$$

where *c* is the interception and *k*_*p*_ is the intraparticle diffusion rate constant which are obtained from the linear plot of uptake (*q*_*t*_) versus the square root of time (*t* ^1/2^) which is shown in Figures 20 and 21. The interception shows the boundary layer thickness; that is, the larger the interception, the greater the boundary layer effect. The calculated intraparticle diffusion coefficient *k*_*p*_ values are listed in Tables 14 and 15.

If the *q*_*t*_ versus *t* ^1/2^ plot is linear and passes through the origin, then only the intraparticle diffusion is the rate controlling mechanism. Otherwise, some other mechanisms along with intraparticle diffusion are also involved [60]. As can be seen from Figures 20 and 21, the interception of the line does not pass through the origin showing that the mechanism of adsorption is not solely govern ed by intraparticle diffusion process.

To investigate the slow step in the adsorption process, the kinetic data were further studied using the Boyd model given by [38]

(14) $$\begin{array}{c} F=1-\frac{6}{\pi^{2}}\exp\left(-B_{t}\right), \end{array}$$

(15) $$\begin{array}{c} F=\frac{q_{t}}{q_{e}}, \end{array}$$

where *q*_*e*_ (mg/g) is the adsorption capacity at the equilibrium time and *q*_*t*_ (mg/g) is the adsorption capacity at any time *t*. *F* is the fraction of solute adsorbed at any time *t* and *B*_*t*_ is a mathematical function of *F*.

Solving the above two equations (15) and (16) we get

(16) $$\begin{array}{c} B_{t}=-0.4977-\ln\left(1-F\right). \end{array}$$

**Figure 22:**
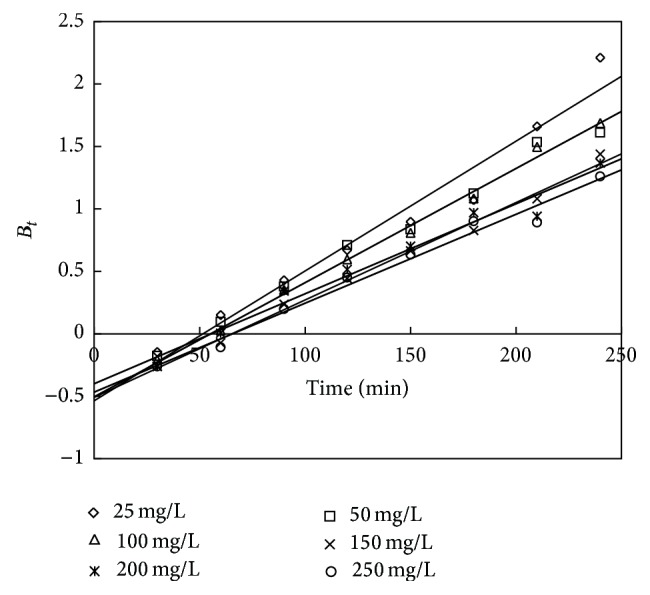
Boyd plot for adsorption of phenol onto adsorbent treated with HCl.

**Figure 23:**
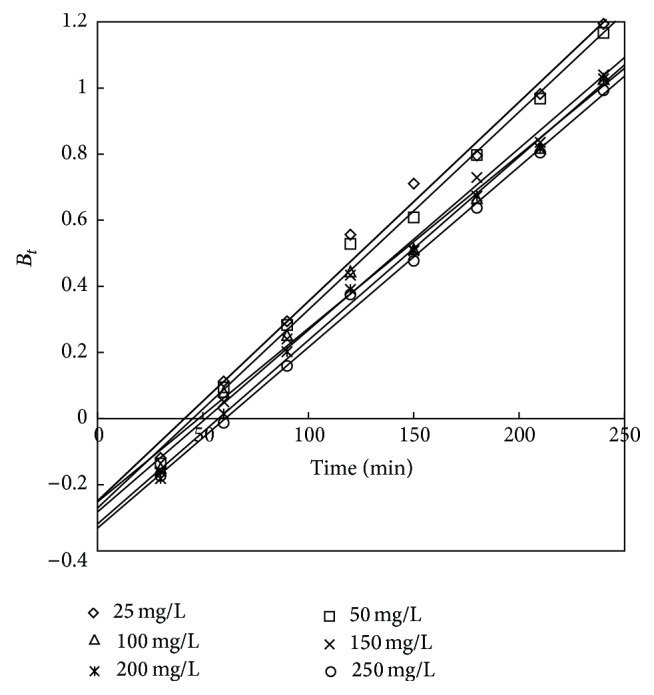
Boyd plot for adsorption of phenol onto adsorbent treated with KOH.

The *B*_*t*_ values were plotted against time *t*, as shown in Figures 22 and 23 for adsorbent treated with HCl and KOH, respectively. The linear lines for all concentrations did not pass through the origin showing that the adsorption of phenol on the chemically treated carbon was mainly governed by external mass transport where particle diffusion was the slowest step.

## 4. Conclusions

The current study shows that* Lantana camara* can be used as an effective adsorbent for the removal of phenol from aqueous solution. The proximate analysis and the estimation of various properties like specific surface area, pore volume, and average particle size signify the effectiveness of the adsorbent. The FTIR study revealed the types of chemical bonds responsible for adsorption. It was found that the amount of phenol adsorbed depended on the parameters like adsorbent dosage, initial dye concentration, pH, and temperature. The rate of adsorption followed pseudo-second-order kinetics model with little deviation of the experimental values from the calculated values. The equilibrium data conform to the Langmuir isotherm equation with the monolayer adsorption capacity of 112.5 mg/g and 91.07 mg/g for adsorbent treated with HCl and KOH, respectively. The determination of thermodynamic parameters shows that the adsorption process is feasible, spontaneous, and exothermic in nature. From the results obtained, the credibility of this forest waste as one of the most suitable precursors for the preparation of adsorbent for pollutant removal has enhanced manifold.

## Figures and Tables

**Table 1:** The concentration of phenol in wastewater released from various industries.

Industrial source	Phenol concentration, mg/L
Petroleum refineries	40–185
Petrochemical	200–1220
Textile	100–150
Leather	4.4–5.5
Coke ovens	600–3900
Coal conversion	1700–7000
Ferrous industry	5.6–9.1
Rubber industry	3–10
Pulp and paper industry	22
Wood preserving industry	50–953
Phenolic resin production	1600
Phenolic resin	1270–1345
Fiberglass manufacturing	40–2564
Paint manufacturing	1.1

**Table 2:** The proximate analysis of the untreated carbon.

Parameter	Value (%)
Volatile matter	46.66
Moisture	6.66
Ash	5.229
Fixed carbon	41.451

**Table 3:** The removal capacity, average particle size, pore volume, and the specific surface area for various carbons.

		Chemically treated carbons					
	Untreated	H_3_PO_4_	KNO_3_	H_2_SO_4_	ZnCl_2_	HCl	KOH
Particle size, *µ*m	23.86	17.98	16.19	19.71	14.22	11.59	11.68
Specific surface area, m^2^/g	115.15	109.90	210.59	170.71	206.65	349.56	328.72
Pore volume, m^3^/g	0.1113	0.1305	0.1789	0.1716	0.1392	0.2780	0.2761
% Phenol removal	68.9	72.6	82.3	84.1	76.2	94.4	95.2

**Table 4:** The FTIR spectral analysis of adsorbent treated with HCl.

Peak	Frequency (cm^−1^)		Difference	Assignment
	Before adsorption	After adsorption		
1	3620	3610	−10	O–H stretching in phenol
2	1203	1218	−15	C–O group in alcohol
3	1689	1697	+8	C=O stretch of carboxylic acid
4	1558	1566	+8	C=C bond of aromatic ring

**Table 5:** The FTIR spectral analysis of adsorbent treated with KOH.

Peak	Frequency (cm^−1^)		Difference	Assignment
	Before adsorption	After adsorption		
1	3610	3633	+23	O–H stretching in phenol
2	1365	1362	−3	C–O ring of alcohol
3	2923	2920	−3	C–H stretching of alkane group
4	1743	1712	−31	C=O in carboxylic group

**Table 6:** Table showing the nature of the process depending on the value of separation factor (*R*_*L*_).

*R*_*L*_ > 1	Unfavourable
*R*_*L*_ = 1	Linear
0 < *R*_*L*_ < 1	Favourable
*R*_*L*_ = 0	Irreversible

**Table 7:** The various parameters and the model equation for adsorbent treated with HCl.

Isotherm model	Model parameter	*R* ^2^	Model equation
Langmuir	*Q*_*m*_ = 112.5 *K*_*f*_ = 0.03951	0.9955	$q_{e}=\frac{4.37C_{e}}{1+0.03851C_{e}}$

Freundlich	*K*_*f*_ = 1.3468 *n* = 5.048	0.9869	*q*_*e*_ = 1.3468*C*_*e*_^0.198^

Temkin	*A* = 0.7471 *B* = 17.67	0.91827	*q*_*e*_ = 17.67ln⁡(0.7471*C*_*e*_)

D-R	*Q*_*m*_ = 60 Beta = 2 × 10^−5^ *E* = 158.22	0.9323	*q*_*e*_ = 60⁡∗*e* ^−(2 × 10^−5^∗*ε*^2^)^

**Table 8:** The various parameters and the model equation for adsorbent treated with HCl.

Isotherm model	Model parameter	*R* ^2^	Model equation
Langmuir	*Q*_*m*_ = 91.07 *K*_*f*_ = 0.0386	0.9964	$q_{e}=\frac{3.515C_{e}}{1+0.0386C_{e}}$

Freundlich	*K*_*f*_ = 1.326 *n* = 4.198	0.9811	*q*_*e*_ = 1.326*C*_*e*_^0.2382^

Temkin	*A* = 0.852 *B* = 13.194	0.90717	*q*_*e*_ = 13.194ln⁡(0.852*C*_*e*_)

D-R	*Q*_*m*_ = 46 Beta = 0.85 × 10^−5^ *E* = 242.535	0.9283	*q*_*e*_ = 46⁡∗*e* ^−(0.85 × 10^−5^∗*ε*^2^)^

**Table 9:** Comparison of monolayer adsorption capacity for phenol onto other various adsorbents.

Adsorbent	*q*_*m*_ (mg/g)	Reference
Date stones	90.3	[17]
*Tamarindus indica *	80	[18]
Vegetal cords	6.21	[19]
Banana peel	688.9	[20]
Palm seed coat	18.3	[21]
Oil palm empty fruit bunch	4.868	[22]
Date pit	262.3	[23]
Black stone cherries	133.33	[24]
Vetiver roots	145	[25]
Sugarcane bagasse	35.71	[26]
*Luffa cylindrica *	9.25	[27]
*Lantana camara* (HCl treated)	112.5	Present work
*Lantana camara* (KOH treated)	91.07	Present work

**Table 10:** The determined thermodynamic parameters for adsorbent treated with HCl.

Conc.	Δ*G*° (J/mol)	Δ*G*° (J/mol)	Δ*G*° (J/mol)	Δ*G*° (J/mol)	Δ*H*° (J/mol)	Δ*S*° (J/mol K)
mg/L	298 K	308 K	318 K	328 K		
25	−7077.08	−6781.68	−6486.28	−6190.88	−15880.00	−29.54
50	−6623.08	−6357.68	−6092.28	−5826.88	−14532.00	−26.54
100	−6248.73	−6046.07	−5843.41	−5640.75	−12288.00	−20.266
150	−5748.2	−5557.2	−5366.2	−5175.2	−11440.00	−19.1
200	−5339.3	−5167.8	−4996.3	−4824.8	−10450.00	−17.15
250	−5264.41	−5101.37	−4938.33	−4775.29	−10123.00	−16.304

**Table 11:** The determined thermodynamic parameters for adsorbent treated with KOH.

Conc.	Δ*G*° (J/mol)	Δ*G*° (J/mol)	Δ*G*° (J/mol)	Δ*G*° (J/mol)	Δ*H*° (J/mol)	Δ*S*° (J/mol K)
mg/L	298 K	308 K	318 K	328 K		
25	−7352.88	−7038.48	−6724.08	−6409.68	−16722	−31.44
50	−6901.54	−6703.84	−6506.14	−6308.44	−12793	−19.77
100	−6548.55	−6432.63	−6316.71	−6200.79	−10003	−11.5921
150	−5976.61	−5871.5	−5766.4	−5661.29	−9108.8	−10.5107
200	−5738.52	−5640.32	−5542.13	−5443.94	−8664.7	−9.8194
250	−5498.06	−5415.18	−5332.29	−5249.41	−7968	−8.28839

**Table 12:** The kinetic constants of first-order and second-order for adsorbent (HCl treated).

Conc. (mg/L)	First-order kinetic				Second-order kinetic			
	*Q*_*e*,exp⁡_ (mg/g)	*K*_1_∗10^3^ (min^−1^)	*Q*_*e*,cal_ (mg/g)	*R* ^2^	*Q*_*e*,cal_ (mg/g)	*K*_2_∗10^4^ (g/mg·min)	*h* (mg/g·min)	*R* ^2^
25	6.2933	9.78	7.3329	0.96574	6.82	24.90	0.1158	0.99732
50	12.48	8.5211	13.0785	0.9729	14.16	7.98	0.1594	0.99327
100	24.64	10.20	33.148	0.95395	33.79	2.26	0.2580	0.99569
150	36.56	9.9489	51.394	0.97226	46.30	1.65	0.3537	0.987
200	47.89	9.327	60.52	0.96477	56.445	1.50	0.4779	0.99053
250	59.7	11.51	73.9	0.9541	64.3	1.48	0.6119	0.9898

**Table 13:** The kinetic constants of first-order and second-order for adsorbent (KOH treated).

Conc. (mg/L)	First-order kinetic				Second-order kinetic			
	*Q*_*e*,exp⁡_ (mg/g)	*K*_1_∗10^3^ (min^−1^)	*Q*_*e*,cal_ (mg/g)	*R* ^2^	*Q*_*e*,cal_ (mg/g)	*K*_2_∗10^3^ (g/mg·min)	*h* (mg/g·min)	*R* ^2^
25	4.76	6.49	3.8939	0.98778	5.27	2.23	0.06193	0.9958
50	9.418	6.07	7.5262	0.98794	10.593	1.024	0.1149	0.9939
100	18.65	6.033	15.85	0.96661	20.24	0.540	0.2212	0.9916
150	27.51	5.987	23.36	0.98688	29.152	0.3617	0.3073	0.9913
200	36.44	6.033	32.32	0.9836	37.9836	0.246	0.3549	0.9925
250	45.28	5.941	40.49	0.98846	47.347	0.1733	0.38838	0.9944

**Table 14:** The kinetics constants of intraparticle diffusion of adsorbent (HCl treated).

Conc. (mg/L)	Intraparticle diffusion		
	*K*_*i*_ (mg/g·min^0.5^)	*c*	*R* ^2^
25	0.31785	0.63245	0.94971
50	0.62941	1.0110	0.94412
100	1.34986	1.0986	0.95896
150	2.0777	1.210	0.94795
200	2.688	1.293	0.95319
250	3.31	1.3514	0.96728

**Table 15:** The kinetics constants of intraparticle diffusion of adsorbent (KOH treated).

Conc. (mg/L)	Intraparticle diffusion		
	*K*_*i*_ (mg/g·min^0.5^)	*c*	*R* ^2^
25	0.22104	0.473	0.97884
50	0.4467	0.752	0.98516
100	0.873	1.226	0.98991
150	1.361	1.284	0.99031
200	1.78213	1.37983	0.99469
250	2.21113	1.39943	0.99499
